# Feasibility of a Pilot Randomized Controlled Trial Examining a Multidimensional Intervention in Women with Gynecological Cancer at Risk of Lymphedema

**DOI:** 10.3390/curroncol28010048

**Published:** 2021-01-13

**Authors:** Shirin M. Shallwani, Anna Towers, Anne Newman, Shannon Salvador, Angela Yung, Lucy Gilbert, Walter H. Gotlieb, Xing Zeng, Doneal Thomas

**Affiliations:** 1 Lymphedema Program, McGill University Health Centre, Montreal, QC H4A 3S5, Canada; anna.towers@mcgill.ca (A.T.); annewman514@gmail.com (A.N.); yung.angela@gmail.com (A.Y.); doneal.thomas@mail.mcgill.ca (D.T.); 2 Physiotherapy Department, McGill University Health Centre, Montreal, QC H4A 3J1, Canada; 3 School of Rehabilitation Sciences, University of Ottawa, Ottawa, ON K1H 8M5, Canada; 4 Department of Oncology, McGill University, Montreal, QC H4A 3T2, Canada; 5 Gynecologic Oncology Program, Jewish General Hospital, Montreal, QC H3T 1E2, Canada; shannon.salvador@mcgill.ca (S.S.); walter.gotlieb@mcgill.ca (W.H.G.); 6 Department of Obstetrics and Gynecology, McGill University, Montreal, QC H4A 3J1, Canada; lucy.gilbert@mcgill.ca; 7 Gynecologic Oncology Division, McGill University Health Centre, Montreal, QC H4A 3J1, Canada; xing.zeng@mcgill.ca

**Keywords:** gynecological cancer, lymphedema, edema, compression, exercise, physical activity, cellulitis, feasibility

## Abstract

There is limited knowledge on non-invasive lymphedema risk-reduction strategies for women with gynecological cancer. Understanding factors influencing the feasibility of randomized controlled trials (RCTs) can guide future research. Our objectives are to report on the design and feasibility of a pilot RCT examining a tailored multidimensional intervention in women treated for gynecological cancer at risk of lymphedema and to explore the preliminary effectiveness of the intervention on lymphedema incidence at 12 months. In this pilot single-blinded, parallel-group, multi-centre RCT, women with newly diagnosed gynecological cancer were randomized to receive post-operative compression stockings and individualized exercise education (intervention group: IG) or education on lymphedema risk-reduction alone (control group: CG). Rates of recruitment, retention and assessment completion were recorded. Intervention safety and feasibility were tracked by monitoring adverse events and adherence. Clinical outcomes were evaluated over 12 months: presence of lymphedema, circumferential and volume measures, body composition and quality of life. Fifty-one women were recruited and 36 received the assigned intervention. Rates of recruitment and 12-month retention were 47% and 78%, respectively. Two participants experienced post-operative cellulitis, prior to intervention delivery. At three and six months post-operatively, 67% and 63% of the IG used compression ≥42 h/week, while 56% engaged in ≥150 weekly minutes of moderate-vigorous exercise. The cumulative incidence of lymphedema at 12 months was 31% in the CG and 31.9% in the IG (*p* = 0.88). In affected participants, lymphedema developed after a median time of 3.2 months (range, 2.7–5.9) in the CG vs. 8.8 months (range, 2.9–11.8) in the IG. Conducting research trials exploring lymphedema risk-reduction strategies in gynecological cancer is feasible but challenging. A tailored intervention of compression and exercise is safe and feasible in this population and may delay the onset of lymphedema. Further research is warranted to establish the role of these strategies in reducing the risk of lymphedema for the gynecological cancer population.

## 1. Introduction

Gynecological cancers comprise over 10% of new cancer cases affecting females and over 11,000 Canadian women were estimated to be diagnosed in 2019 alone [1]. Lymphedema, a chronic inflammatory state with irreversible accumulation of protein-rich lymph fluid in the interstitial tissues, is a common side effect of surgery and radiation therapy in patients with cancer. The incidence of lower limb lymphedema in patients treated for gynecological cancers has been found to range from 20% to 45% [2,3,4]. Studies have demonstrated that while the majority of cases occur in the first year, the risk of developing lymphedema after gynecological cancer persists in the long term [4,5]. Disease and treatment-related risk factors for the development of gynecological cancer-related lymphedema include: diagnosis of vulvar cancer, surgical lymph node dissection, radiation therapy, adjuvant chemotherapy, and the presence of post-operative complications such as lymph cysts, lymphoceles and local infections [2,6,7,8,9,10,11,12,13,14,15]. Additional risk factors include obesity, older age and pre-treatment lymphedema [6,9,15].

Gynecological cancer-related lymphedema remains highly under-recognized and under-treated, in part due to a lack of awareness among health professionals and patients regarding early diagnosis and treatment of leg edema, an absence of standardized evaluation and diagnostic methods and a shortage of certified lymphedema specialists [2,6,9,16,17]. These challenges commonly result in delayed diagnosis and late referral to specialized lymphedema services. Characterized by chronic swelling, inflammation and fibrosis, lymphedema can also predispose patients to recurrent bacterial cellulitis, which may be potentially life-threatening [18]. Lower limb lymphedema after treatment for gynecological cancer has been associated with persistent symptoms (e.g., achiness and pain), poor sleep satisfaction, issues with appearance and self-image and financial problems [19,20,21,22]. Moreover, patients with chronic edema have reported difficulties with mobility, housework, physical activity, employment and social activities, along with challenges related to skin infections, antibiotic use and hospitalizations [23]. Decongestive therapies, particularly compression therapy, have been found to be beneficial in the management of lower limb lymphedema in terms of limb volume reduction and improvement of physical functioning [24,25,26,27]. However, treatment techniques are costly, requiring time and financial resources for frequent therapist sessions and compression materials [28,29].

Since lymphedema imposes tremendous physical and psychosocial challenges, further attention is needed to define risk reduction strategies, particularly in the gynecological cancer population. Currently, most research has focused on newer and more conservative surgical techniques, such as sentinel lymph node biopsy and laparoscopic approaches, and materials such as fibrin sealants [17]. Physical decongestive strategies, such as compression therapy and exercise, have been found to be effective for the management of lymphedema [30]. However, their role in reducing the risk of lymphedema development has not been well explored. To address these questions and to guide future research efforts, pilot randomized controlled trials (RCTs) are useful. They allow us to explore feasibility outcomes and to examine the potential effectiveness of novel interventions [31,32]. The objectives of this report are: (a) to describe the design of a pilot RCT examining a tailored multidimensional intervention in women treated for gynecological cancer at risk of developing lymphedema; (b) to report on feasibility of the study procedures; including participant recruitment, randomization and retention, outcome assessment procedures, as well as the delivery and components of the tailored intervention; and (c) to explore the preliminary effectiveness of the intervention on the cumulative incidence of lymphedema at 12 months.

## 2. Materials and Methods

### 2.1. Study Design and Setting

This study was a pilot single-blinded, parallel-group, multi-centre RCT (ClinicalTrials.gov Identifier: NCT02966327) that occurred from January 2015 to April 2019. We aimed to recruit 50 women with newly diagnosed gynecological cancer pre-operatively from departments at two hospital sites located in Montreal, Canada: the Gynecologic Oncology Division at the McGill University Health Centre (MUHC) Royal Victoria Hospital and the Gynecologic Oncology Program at the Jewish General Hospital (JGH) Segal Cancer Centre. Study participants were stratified post-operatively according to radiation therapy status and randomized in a 1:1 ratio using a computer-generated randomizer to either an intervention group (IG) or a control group (CG). The study assessments and intervention delivery occurred at the MUHC Lymphedema Clinic. The primary clinical outcomes were assessed by lymphedema-specialized clinicians blinded to group allocation. Blinding of the study participants and the therapist delivering the interventions was not possible due to the nature of the study interventions. Ethics approval for this study was obtained from the McGill University Research Ethics Board.

### 2.2. Participants

Eligible participants were identified by the treating oncologists and surgeons or the clinic nurse coordinators at the two hospital clinics and were contacted for recruitment by the study coordinator. Inclusion criteria were: (1) ≥18 years of age; (2) initial diagnosis of: (a) grade 2 or 3 endometrial cancer, or high-grade type (serous or clear cell); (b) stage 1b1 or stage 2a cervical cancer; or (c) stage 1 vulvar cancer with tumor greater than 4 cm, stage 2 or 3 vulvar cancer and (3) planned to undergo surgical lymph node dissection. The following exclusion criteria were applied: (1) diagnosis of recurrent gynecological cancer; (2) stage 4 cancer; (3) BMI > 40; (4) presence of pre-existing lymphedema; (5) sentinel lymph node dissection only.

### 2.3. Sample Size Justification

The design of this pilot study was aimed at assessing feasibility and hence no minimum sample size calculation was performed. Based on review of previous pilot studies and knowledge of clinical caseloads within our oncology and lymphedema clinics, we projected an enrollment of 50 participants would be sufficient to assess the feasibility of conducting the study and delivering the multidimensional intervention within a reasonable timeframe. The estimates derived from this pilot study would provide adequate information, including anticipated level of precision and power, to perform sample size calculations for subsequent studies.

### 2.4. Intervention

At four to six weeks post-operatively (T2), participants in both the CG and IG received standard education on lymphedema risk reduction by a lymphedema therapist non-blinded to group allocation, along with printed English or French information materials developed by the Lymphedema Association of Quebec. At this time, each participant in the IG was prescribed standard or custom-made compression class 1 (18–21 mmHg) stockings for bilateral lower limbs with or without panty, depending on personal preference and recommendation by the garment fitter. A certified garment fitter with specialized training in lymphedema care performed the limb measurements for the compression stockings at each participant’s home following the T2 clinic visit and supplied the garments at no cost to the participants. Participants were asked to wear the stockings for 12 to 16 h daily for at least six months post-operatively. Participants in the IG also received individualized education from the certified lymphedema therapist on general aerobic and resistance exercise according to the Canadian Physical Activity Guidelines [33], self-lymphatic drainage (Casley-Smith method [34]) and skin care.

Participants in the CG were only prescribed compression stockings as part of standard lymphedema therapy if lymphedema developed over the course of the study. Any participant enrolled in the study presenting with stage 2 or 3 lymphedema was referred to a hospital-based lymphedema therapist for complete decongestive therapy as part of standard care.

### 2.5. Outcome Measures

Study participants were evaluated at five time points: (a) pre-operatively (T1); (b) four to six weeks post-operatively (T2); (c) three months post-operatively (T3); (d) six months post-operatively (T4); and (e) 12 months post-operatively (T5). Baseline descriptive variables, including demographic factors (age, marital status, employment status), medical history (BMI, comorbidities) and cancer history (cancer diagnosis, treatment protocol), were collected at T1 and T2. Feasibility outcomes were measured by tracking the recruitment period and rates of recruitment (number of patients consenting to participate pre-operatively/number referred to study) and retention (number of participants completing 12-month study/number receiving allocated intervention post-operatively). Reasons for exclusion were recorded over the course of the study. The participant flow through the study phases was reported according to the CONSORT 2010 Statement [35]. Feasibility of the study assessment procedures was measured by tracking the rates of completion for each outcome measure at each study time point. Intervention safety was assessed by monitoring serious adverse events (e.g., cellulitis) at each time point. Intervention feasibility was evaluated based on reported use of compression (average number of hours of daily use in last week) in the IG at T3 and T4 and reported weekly levels of exercise for both groups at the five study time points. Adherence to the intervention was determined in the IG participants based on the use of compression for at least 35 h per week and participation in at least 150 min of moderate and/or vigorous exercise per week at the T3 and T4 visits.

At each of the five study time points, clinical outcomes were assessed by a physician and a physiotherapist who were specialized in lymphedema therapy. These clinicians were blinded to group allocation during the study assessments.

- Presence and characteristics of lymphedema: The diagnosis of lymphedema was based on clinical examination. Specifically, the presence of soft pitting edema or fibrotic non-pitting edema along the lower limbs and/or the presence of a positive Stemmer sign (thickened skin fold at base of second toe) were evaluated [17,36,37,38]. Lymphedema was staged according to guidelines provided by the International Society of Lymphology 2013 Consensus Document [39].
- Lower limb circumferential measures: Bilateral lower limb circumferential measures were obtained using a tape measure and a measurement board. Participants were in a supine position and the lower limb was placed on the measurement board. Serial circumferential measures to the nearest millimeter were taken with a retractable no-stretch soft tape measure at eight points along each limb: (1) 10 cm from heel around foot; (2) superior to malleoli (about 10 cm from heel); (3) 10 cm above second point; (4) 20 cm above second point; (5) 30 cm above second point; (6) 40 cm above second point; (7) 50 cm above second point; (8) groin. Total and segmental limb volumes for each limb were calculated using the truncated cone method [40].
- Lower limb volume: Total and segmental volume measurements of the bilateral lower limbs were measured with an optoelectronic infra-red volumeter (perometry) (Pero-System Messgeraete GmbH, Wuppertal, Germany, Perometer 350S) [41]. The perometry device was placed on an adjustable table and participants were seated on an adjacent chair with adjustable height. The lower limb was extended with the foot supported so the limb was parallel to the device for measurement. The volume of each limb was measured once, by moving the frame of the device slowly along the limb.

In addition, the following clinical outcomes were collected by the study coordinator, who was non-blinded to group allocation.

- Body composition: Changes in intracellular and extracellular lower limb fluid were measured by assessing ratios of resistance at infinite (Ri) to resistance at zero (Ro) through bioimpedance spectroscopy (ImpediMed Ltd., Carlsbad, CA, USA, Imp SFB7) [42]. The bioimpedance measurements were obtained using a tetrapolar surface electrode arrangement. Participants were supine and after cleansing the skin surface, four single-use surface electrodes were placed with reference to anatomical markers: two drive electrodes were placed on the dorsal surface of the hand, 1 cm proximal to the metacarpophalangeal joint of the middle finger, and on the mid dorsum of the foot 1 cm proximal to the metatarsophalangeal joint of the second toe; two measurement (voltage-sensing) electrodes were placed on the dorsal surface of each ankle between the medial and lateral malleoli of the ankle. This procedure was repeated on both limbs. Measurements began within five minutes of lying down and were completed within ten minutes. All but two measurements were taken in the morning between 8:00 a.m. and 10:00 a.m.
- Quality of life (QOL): Patient-reported QOL was measured by administering the 30-item European Organization for Research and Treatment of Cancer (EORTC) Core Quality of Life Questionnaire (QLQ-C30) (EORTC Quality of Life Group, version 3.0). This 30-item questionnaire yields five functional subscale scores, nine symptom subscale scores and one global health status score [43].

For the purpose of this report on the feasibility of the pilot RCT, data are reported on the primary clinical outcome of lymphedema development. The presence of lower limb lymphedema was confirmed based on clinical observation and physical examination (e.g., pitting test, Stemmer sign) by lymphedema-specialized health professionals blinded to group allocation during the study assessments.

### 2.6. Statistical Analysis

Baseline data were described by calculation of frequency (n) and relative frequencies (%), mean, standard deviation (sd) and range. Between-group homogeneity was analyzed by Chi-square and Fisher’s exact tests for categorical variables and the Mann–Whitney tests for continuous variables. The level of significance considered was a two-sided *p*-value less then 0.05. Time to lymphedema diagnosis was calculated according to the number of months between surgery and the time point when lymphedema presence was confirmed. The cumulative incidence of lymphedema stratified by group was calculated using the Kaplan-Meier method to demonstrate the probability of lymphedema development during the 12-month follow-up and compared between the study groups using the log-rank test. All statistical analyses were conducted using the R statistical software (The R Foundation for Statistical Computing, Vienna, Austria, version 3.6.1).

## 3. Results

### 3.1. Recruitment and Retention

**Figure 1:**
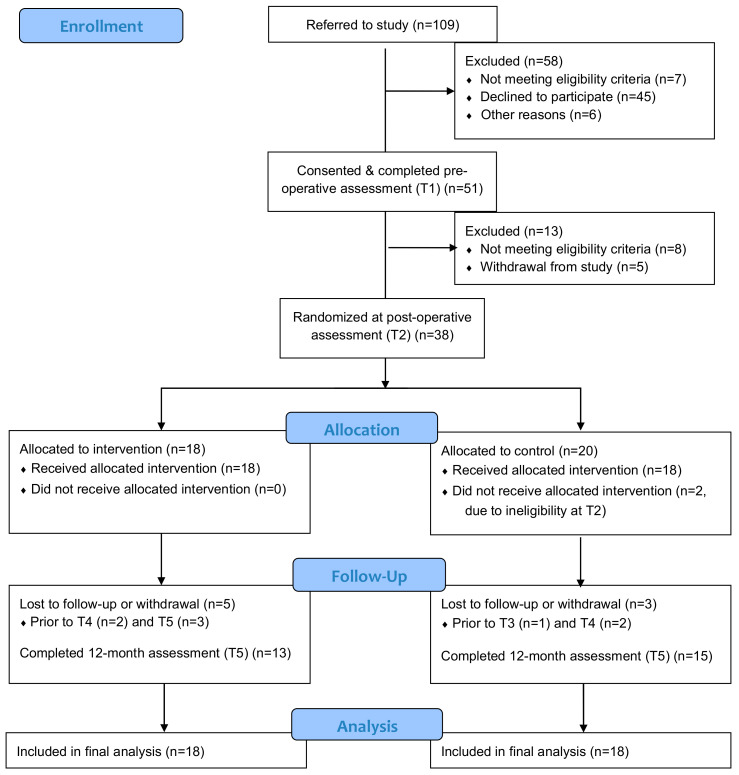
CONSORT 2010 Flow Diagram.

The flow diagram for the study participants is provided in Figure 1.

The recruitment period was 30 months (2015–2018) and 51 of 109 referred patients consented to participate in the study (47% recruitment rate). Post-operatively, 38 eligible participants were randomized to the study groups, with two additional participants in the CG being excluded following the T2 assessment. Thus, 36 participants received the allocated interventions and were included in the analysis. Reasons for exclusion from pre-operative recruitment (*n* = 51) to post-operative intervention delivery (*n* = 36) included withdrawal from the study (*n* = 5), the presence of pre-existing conditions (e.g., obesity, limb edema) or other complications (e.g., metastatic cancer, hospitalization) (*n* = 5), sentinel lymph node dissection instead of standard node dissection (*n* = 4) and no surgical procedure (*n* = 1). Of the 36 participants who received the interventions and were included in the analysis, 28 completed the 12-month protocol (78% retention rate). Baseline characteristics of the 36 participants included in the final analysis are provided in Table 1. Baseline characteristics in the randomized groups were well balanced with no statistically significant differences between the IG and the CG (*p* > 0.05).

### 3.2. Outcome Assessment Procedures

The number of participants completing each outcome measure is provided in Table 2 for each study time point. For those participants who were able to be evaluated at each time point, the highest rates of completion were for the clinical examination (mean over five study visits: 100%), the circumferential measures (99.6%) and the QOL questionnaire (96.1%), while the lowest were for perometry (82.4%) and bioimpedance spectroscopy (88.7%).

### 3.3. Intervention Safety and Feasibility

Regarding safety, two participants (one in the CG and one in the IG) developed cellulitis, with both episodes occurring post-operatively prior to the participants receiving the allocated study intervention at T2. No other serious adverse effects were reported.

Of 18 participants in the IG, three participants received individualized education on exercise and lymphedema risk reduction at the post-operative visit (T2) but did not obtain compression garments from the fitter following the clinic visit. The remaining 15 participants received standard compression stockings, except one who required a custom-made garment. Within these 15 participants, six participants received compression stockings with attached panty, eight participants each received a pair of thigh-high stockings, and one participant received one garment of each type. At T3 and T4, 67% (*n* = 12/18) and 63% (*n* = 10/16) of the IG participants reported using compression therapy for at least 42 h per week. Factors that were reported by the participants to limit their adherence to compression therapy included discomfort, body image issues, chemotherapy-related limb pain, and inability to don garments due to physical limitations.

Rates of participation in at least 150 min per week of moderate- to high-intensity exercise over the 12-month study are demonstrated in Table 3. Reported barriers to engaging in physical activity included fatigue, weakness, pain and lack of time.

### 3.4. Preliminary Effectiveness on Lymphedema Incidence

**Figure 2:**
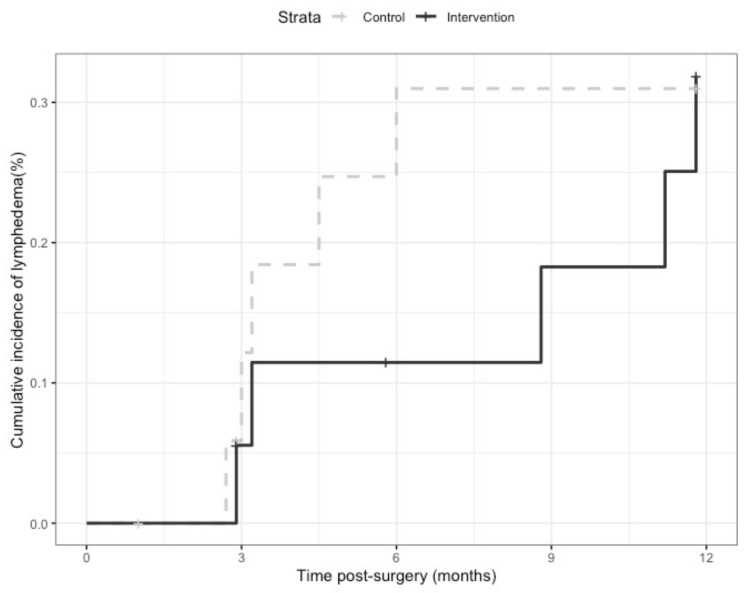
Cumulative incidence of lymphedema by intervention group and control group.

The cumulative incidence of lymphedema at 12 months post-operatively was 31% (*n* = 5 cases) in the CG and 31.9% (*n* = 5 cases) in the IG (Figure 2, log-rank *p* = 0.88). At three and six months post-operatively, the cumulative incidence rates were 12.2% and 31% in the CG and 5.6% and 11.5% in the IG, respectively. In the 10 participants who developed lymphedema, the median time to lymphedema diagnosis was 3.2 months (range, 2.7–5.9) in the CG vs. 8.8 months (range, 2.9–11.8) in the IG.

## 4. Discussion

To our knowledge, this is the first trial exploring a multidimensional intervention of compression therapy and individualized exercise education in women treated for gynecological cancer at risk of developing lymphedema. Our findings support the feasibility of participant recruitment, randomization, and retention, the outcome assessment methods and the intervention delivery within this pilot RCT. While the tailored intervention of combined compression and exercise does not appear to reduce the incidence of lymphedema in people with gynecological cancer, it may delay the onset of lymphedema in this population. Challenges and facilitators to conducting this trial have been highlighted, as well as suggestions for future research.

### 4.1. Participant Recruitment and Retention

Recruitment and retention to RCTs of this type is feasible but challenging. While our target of recruiting 50 women was met, the recruitment period took longer than originally anticipated and over half of the women referred to the study refused participation. The retention rate (78%) to the study at 12-months was acceptable, particularly considering its long-term timeline. The main reasons for non-recruitment and non-retention included declined or withdrawn participation, ineligibility factors, and incomplete pre-operative assessments. Several patients declined or withdrew participation from the study for reasons including personal stress and lack of time. These may have been related to the introduction of the research study prior to commencing cancer treatment, the frequency and location of the study assessments and the potentially intensive nature of the multidimensional intervention. Additionally, many patients did not meet our eligibility criteria prior to and following recruitment due to pre-existing medical conditions (e.g., morbid obesity, limb edema), complications (e.g., hospitalization, metastatic disease) and changes in surgical procedure (e.g., sentinel lymph node biopsy, no surgery at all). Recent studies have highlighted the high and often unrecognized prevalence of chronic edema in the general population, particularly in people with obesity or other comorbidities [16,44,45]. Moreover, despite our specific eligibility criteria, it was difficult to precisely predict which patients would undergo surgical lymph node dissection due to the pre-operative timing of participant recruitment and the recent shift in surgical practice towards sentinel node biopsy [17]. Finally, a few participants agreed to participate in the study, but underwent surgery prior to completing their pre-operative assessments at the lymphedema clinic. This issue also highlights the challenges associated with recruiting patients and gathering baseline data prior to commencing cancer treatment, as well as with performing the study assessments at a location distant from the site of oncology follow-up.

Facilitators to recruitment and retention to this study included the inclusion of multiple hospital sites, the support and involvement of the referring surgeons and supporting staff (e.g., nurse coordinator, administrative assistant), the automatic referral of patients likely to undergo surgical lymph node dissection (e.g., grade 3 endometrial cancer), the regular on-site presence of the study coordinator, and the accommodation of follow-up study assessments on-site at the hospital cancer clinics. Researchers conducting future trials should consider strategies to minimize patient burden and inconvenience and to facilitate study procedures, such as the coordination of on-site study assessments (pre-operatively in particular) and the integration of referring surgeons and oncology health professionals in the early stages of research planning and implementation.

### 4.2. Outcome Assessment Procedures

Overall, at least 80% of the participants in the study were able to complete all of the outcome assessments at each study time point. In a few cases, in order to prevent dropout, follow-up study assessments were accommodated at a different part of the hospital more convenient to the participant. The highest rates of completion were for the clinical examination, the circumferential measures and the QOL questionnaire. These assessment tools required minimal time, equipment and resources to administer and were possible to complete remotely from the lymphedema clinic. In contrast, the perometry and the bioimpedance spectroscopy assessments had the lowest rates of completion, mainly due to technical or administration issues with these types of equipment and the fixed location of the perometry device at the lymphedema clinic. Our findings demonstrate the challenges of completing comprehensive lymphedema evaluations for research purposes, particularly in real-life clinical settings, and highlight the characteristics of assessment tools that facilitate their administration, namely, ease of use, accessibility and portability. Clinical examinations by lymphedema specialists, consisting of medical history taking, symptom assessment, visual assessment, skin evaluation and palpation, have been found to be accurate and useful for the evaluation of gynecological cancer-related lymphedema [17,46]. Moreover, the validity of volumetric and bioimpedance assessment techniques has been demonstrated in the literature [47]. Given the challenges with standardizing clinical assessment techniques, further work is needed to determine best practices for the evaluation of lower limb lymphedema in research and clinical settings. Recommendations for edema assessment involve standardizing measurement protocols, using blinded methodology, incorporating serial measurements over time (vs. pre- and post-intervention alone), including long-term follow-up assessments and evaluating clinical outcomes related to limb edema (e.g., volume) as well as patient-reported function and symptoms [47].

### 4.3. Intervention Safety and Feasibility

One major issue associated with lymphatic insufficiency is the risk of cellulitis infection, which often requires hospitalization for the intravenous administration of antibiotics and may potentially be life-threatening [18]. In our study, there were two cases of cellulitis, both occurring immediately post-operatively prior to the participants receiving the assigned intervention. This finding is consistent with or lower than rates previously reported in the cancer literature [48,49]. Despite the common occurrence of recurrent cellulitis infections [48], no participant in our trial developed cellulitis after receiving the study or control intervention over the course of the entire 12-month study. It is possible that the education provided on lymphedema risk reduction to both study groups may have been more informative than what is provided as part of usual standard care. The effect of educational interventions may be worth further exploring in future research efforts. No other adverse events were reported in our study, supporting the safety of the multidimensional intervention in our study.

In our pilot trial, two-thirds of the IG participants adhered to using the compression stockings regularly for six months post-operatively. Similarly, Stuiver et al. (2014) reported 24 of 33 evaluable patients with melanoma and urogenital tract wore compression stockings for six months [50]. These rates are lower than those reported by Sawan et al. (2009) in their pilot study of 14 women with vulvar cancer, of which six of seven women adhered to the use of compression stockings post-operatively [51]. Similar to previous studies, factors contributing to non-adherence with compression garments included patient-reported symptoms of discomfort and chemotherapy-related pain, issues with appearance and difficulty with donning garments. In our study, most participants received standard compression stockings. Alternative materials, custom-made designs and donning aids may be beneficial to address challenges in patients demonstrating decreased tolerance to compression. Further research is warranted to examine barriers to compression therapy and to develop strategies to mitigate these issues, in patients with diagnosed lymphedema or at risk of developing lymphedema alike [52,53,54].

Unexpectedly, over half of the participants in our study, including the CG, engaged in exercise at most points throughout the 12-month study (except for post-operatively in the IG). These rates are higher than those reported in the general gynecological cancer population [21,55,56], suggesting a potential volunteer bias in the patients who consented to participate and remained in our study. The participants demonstrated a high interest and knowledge of physical activity at baseline and may have exhibited a desire to learn about conservative strategies to support their post-operative recovery. The finding of similar exercise levels at baseline in both groups further promotes the value of the RCT design to control these types of selection bias. However, future trials should consider recruitment strategies to include a sample that is more representative of the general gynecological cancer population in order to enhance the transferability of research findings to this clinical population. In addition to this selection bias, there may have been benefits with the educational components of both the study and control interventions provided in our trial, contributing to the sustained levels of physical activity over the course of the study. Finally, these findings may have been impacted by the Hawthorne effect, which is behaviour influenced “as a motivational response to the interest, care, or attention received through observation and assessment” [57]. This effect can influence the behaviour of study participants in both groups as well as the health professionals conducting the research assessments and delivering the interventions. In our study, the single-blinded RCT design, the frequency of the follow-up assessments and the nature of the interventions may have affected behaviour in both study groups, including the CG. Through awareness of the trial’s focus and design as well as the acquired knowledge of the lymphedema risk reduction strategies, participants in both groups may have felt motivated to continue engaging in physical activity. Due to the nature of the intervention, it was not possible to blind the study participants or the therapists providing the interventions in our study. However, biases related to the Hawthorne effect can occur even in double-blinded clinical trials or other research designs. Mixed-methods research designs can serve as one approach to address this challenge and to better understand the experiences of participants and professionals within research contexts [57]. These methods have not been widely used in the lymphedema literature and may be worth further exploring.

Our findings support the overall safety and feasibility of the lymphedema risk reduction strategies studied in our trial. However, they also highlight challenges in several study participants with adherence to intensive multidimensional interventions, particularly with a physical activity component. Preferences for physical activity programs reported in earlier studies with the general gynecological cancer population include components of goal-setting, walking activities and weekly follow-ups [58,59]. The benefits of physical activity in survivors of gynecological cancer have been previously highlighted and include improved fatigue, physical function, weight and QOL [21,55,56]. In women with gynecological cancer-related lymphedema, benefits associated with exercise include improved strength and endurance, decreased lower limb volume (short-term) and enhanced physical function [60,61,62]. Future studies exploring lymphedema risk reduction strategies in gynecological cancer may consider the integration of tailored recommendations and frequent follow-ups, as well as a more intensive, developed physical activity component.

### 4.4. Lymphedema Incidence 

In our study targeted to women at risk of developing lymphedema due to surgical lymph node dissection, the cumulative incidence rates of lower limb lymphedema were 31 to 32%. Although a wide range of incidence rates have been reported in the literature [17], our findings are similar to recent estimates of lymphedema incidence in women with gynecological cancer [2,3,4]. This pilot trial was not designed to formally test the intervention effectiveness and therefore, the study sample size was limited. Our analysis of the preliminary effectiveness of the intervention revealed no significant difference in lymphedema rates between the two study groups at 12 months post-operatively. These findings are consistent with other studies examining the use of compression stockings for six months post-operatively in people at risk of developing lymphedema after gynecological cancer [50,51]. Our study intervention also consisted of individualized education on exercise provided by certified lymphedema therapists. Non-invasive strategies, such as prospective surveillance, early physiotherapy and exercise, have shown potential benefits on lymphedema-related outcomes in the breast cancer population [63,64,65,66]. However, in a recent randomized trial of 554 women with breast cancer, a combined intervention of compression sleeve and exercise education did not reduce the incidence of lymphedema at 18 months [67]. In addition to implementing research strategies to support intervention adherence, it may be important to further consider the design of exercise program components, such as professional supervision, gradual exercise progression and the integrated use of technology. Due to the small sample size of our pilot trial, no conclusive evidence can be derived from our findings on the effectiveness of a combined compression and exercise intervention on lymphedema incidence in people with gynecological cancer.

Although there were no differences noted in lymphedema development at 12 months, our exploratory findings demonstrated a trend towards delayed onset of lymphedema in the IG compared to the CG. Cumulative incidence rates at three and six months post-operatively were lower in the IG than in the CG. In the participants who developed lymphedema, the median time to lymphedema diagnosis after surgery was almost three times longer in the IG participants than in the CG. Time to diagnosis was not specifically reported on in previous studies examining similar interventions aimed at preventing cancer-related lymphedema [51,67]. As most cases of gynecological cancer-related lymphedema have been found to occur within the first three to six months of cancer treatment [9], there may be some benefit with delaying the onset and minimizing the progression of lymphedema in order to facilitate its timely and successful management. Compression may improve fluid accumulation by reducing capillary filtration and promoting the resorption of fluid into venous and lymphatic systems, but the role of compression therapy as a lymphedema risk reduction strategy is still not well understood [47,68]. Exercise may further enhance lymphatic drainage and has been found to be safe for people with or at risk of lymphedema, particularly after breast cancer [60,64,66,69,70,71]. In a cross-sectional survey of 213 uterine cancer survivors, increased physical activity levels were associated with lower rates of lymphedema, with a dose-response effect noted [21]. It would be worthwhile to further explore the use of compression therapy, with and without exercise, as well as different exercise parameters and program components in people with gynecological cancer. While these non-invasive strategies may play an important role in people with and at risk of developing cancer-related lymphedema, larger studies are needed to understand their effects on lymphedema-related outcomes specifically in the gynecological cancer population.

## 5. Conclusions

This pilot RCT confirms the feasibility of formally assessing lymphedema risk reduction strategies in women treated for gynecological cancer who are at risk of developing lymphedema. A tailored intervention of compression therapy and individualized exercise is safe and feasible in this population and may delay the onset of lymphedema. Due to the limited sample size of our pilot study, there is no conclusive evidence on the effectiveness of the multidimensional intervention on lymphedema outcomes. Future research efforts should carefully implement additional considerations to enhance the feasibility of conducting such studies and to support adherence to post-operative intervention strategies. Further research is warranted to establish the role of compression and exercise in reducing the risk of lymphedema in the gynecological cancer population.

## Figures and Tables

**Table 1:** Baseline/post-operative characteristics (*n* = 36) ^a^.

Characteristics	Overall	IG	CG
Sample size	36	18	18
Age (years), mean (sd)	57.6 (9.6)	56.3 (10.1)	58.9 (9.1)
Employment status			
Full time	22 (61)	11 (61)	11 (61)
Part time	5 (14)	3 (17)	2 (11)
Retired	8 (22)	3 (17)	5 (28)
Unemployed	1 (3)	1 (6)	0 (0)
Body mass index ^b^ (kg/m^2^)			
Mean (sd)	27.5 (5.9)	27.2 (6.2)	27.9 (5.8)
Range	17.9–40.9	18.5–40.9	17.9–38.5
Body mass index category ^b^			
Underweight	1 (3)	0 (0)	1 (6)
Normal weight	14 (39)	9 (50)	5 (28)
Overweight	9 (25)	5 (28)	4 (22)
Obese	12 (33)	4 (22)	8 (44)
Treatment status			
Radiotherapy	19 (53)	9 (50)	10 (56)
No radiotherapy	17 (47)	9 (50)	8 (44)
Cancer diagnosis			
Endometrial	26 (73)	13 (72)	13 (72)
Cervical	7 (19)	4 (22)	3 (17)
Vulvar	3 (8)	1 (6)	2 (11)
Comorbidities ^c^			
None	12 (33)	7 (39)	5 (28)
One	10 (28)	7 (39)	3 (17)
Two or more	14 (39)	4 (22)	10 (56)
Marital status ^d^			
Married	23 (64)	11 (61)	12 (67)
Other	13 (36)	7 (39)	6 (33)

CG: control group; IG: intervention group. ^a^ Unless otherwise indicated, data are expressed as number (percentage) of patients. Percentages have been rounded and may not total 100. ^b^ Body mass index was calculated as weight (kg)/height^2^ (m) based on patient’s height and weight at baseline: underweight (<18.5), normal weight (18.5 to <25), overweight 25 to <30) and obese (≥ 30). ^c^ Comorbidities were calculated using the following variables: obesity, hypertension, diabetes, cardiovascular disease, thyroid, previous cancer diagnosis, arthritis, chronic swelling and cellulitis. ^d^ Other comprised single status (*n* = 11), divorced (*n* = 1) and widow (*n* = 1).

**Table 2:** Outcome assessment completion rates.

Study Time Point	T1	T2	T3	T4	T5
Number of participants	51	38	35	31	28
Clinical examination	51 (100%)	38 (100%)	35 (100%)	31 (100%)	28 (100%)
Circumferential measures	50 (98%)	38 (100%)	35 (100%)	31 (100%)	28 (100%)
Perometry	43 (84.3%)	31 (81.6%)	29 (82.9%)	24 (77.4%)	24 (85.7%)
BIS	48 (94.1%)	33 (86.8%)	35 (100%)	26 (83.9%)	22 (78.6%)
EORTC-QLQ-C30	51 (100%)	38 (100%)	33 (94.3%)	29 (93.5%)	26 (92.9%)

BIS: bioimpedance spectroscopy. EORTC-QLQ-C30: 30-item European Organization for Research and Treatment of Cancer Core Quality of Life Questionnaire.

**Table 3:** Participation in at least 150 weekly minutes of moderate- to high-intensity exercise.

	T1	T2	T3	T4	T5
CG	62% (*n* = 13/21)	56% (*n* = 10/18)	53% (*n* = 9/17)	60% (*n* = 9/15)	53% (*n* = 8/15)
IG	61% (*n* = 11/18)	28% (*n* = 5/18)	56% (*n* = 10/18)	56% (*n* = 9/16)	69% (*n* = 9/13)

CG: control group; IG: intervention group.

## Data Availability

The data are not publicly available due to containing clinical information that could compromise the privacy of research participants. The data that support the reported findings of this study are available from the corresponding author, S.M.S., upon reasonable request.
